# Supplementary material for: Bone Fusion in Normal and Pathological Development is Constrained by the Network Architecture of the Human Skull
Source: Sci Rep. 2017 Jun 13;7:3376. doi: 10.1038/s41598-017-03196-9 (PMC5469793; doi:10.1038/s41598-017-03196-9)
Supplement: Supplementary file 1 — Supplementary Material [file 41598_2017_3196_MOESM1_ESM.pdf]

Supplementary Information

Bone Fusion in Normal and Pathological Development is Constrained by the Network Architecture of the Human Skull

Borja Esteve-Altava[1,2,§], Toni Vallès Català[3,§], Diego Rasskin-Gutman[4], Roger Guimerà[3,5], and Marta Sales-Pardo[3*]

STable 1. Group descriptives of craniofacial connections fused and persistent.

Group	N	Mean	SD	SE
craniosynostosis	13	0.398	0.088	0.024
normal	80	0.481	0.168	0.019

STable 2. Mann-Whitney U Test of fused vs. persistent craniofacial connections.

						95% Confidence Internval	
	**W**	***p-value***	**Mean difference**	**SE difference**	**Cohen's *d***	**Lower**	**Upper**
Score	368	0.047	-0.087	0.031	-0.52	-∞	-0.001

STable 3. Group descriptives of craniofacial sutures affected by craniosynostosis and non-affected.

Group	N	Mean	SD	SE
craniosynostosis	6	0.313	0.104	0.042
normal	87	0.48	0.159	0.017

STable 4. Mann-Whitney U Test of craniofacial sutures affected by craniosynostosis and non-affected.

						95% Confidence Internval	
	**W**	***p-value***	**Mean difference**	**SE difference**	**Cohen's *d***	**Lower**	**Upper**
Score	98	0.006	-0.169	0.046	-1.066	-∞	-0.064

STable 5. Group descriptives of craniofacial sutures affected by craniosynostosis and fused normally.

Group	N	Mean	SD	SE
craniosynostosis	6	0.313	0.104	0.042
normal	11	0.407	0.094	0.028

STable 6. Mann-Whitney U Test of craniofacial sutures affected by craniosynostosis and fused normally.

						95% Confidence Internval	
	**W**	***p-value***	**Mean difference**	**SE difference**	**Cohen's *d***	**Lower**	**Upper**
Score	15.50	0.087	-0.092	0.051	-0.964	-0.235	0.045
